# Segmental Arterial Mediolysis

**DOI:** 10.5334/jbsr.2470

**Published:** 2021-05-20

**Authors:** Gerald Gheysens, Jan Van Ballaer, Geert Maleux

**Affiliations:** 1 UZ Leuven, BE; 2 RZ Tienen, BE

**Keywords:** Computed tomography, vasculopathy, dissection, occlusion, haemorrhage, acute abdomen

## Abstract

**Teaching Point:** Segmental arterial mediolysis is a rare cause of acute abdominal pain due to dissection and/or aneurysm formation in visceral arteries with subsequent stenosis, occlusion, or haemorrhage.

## Case Study

**Figure 1:**
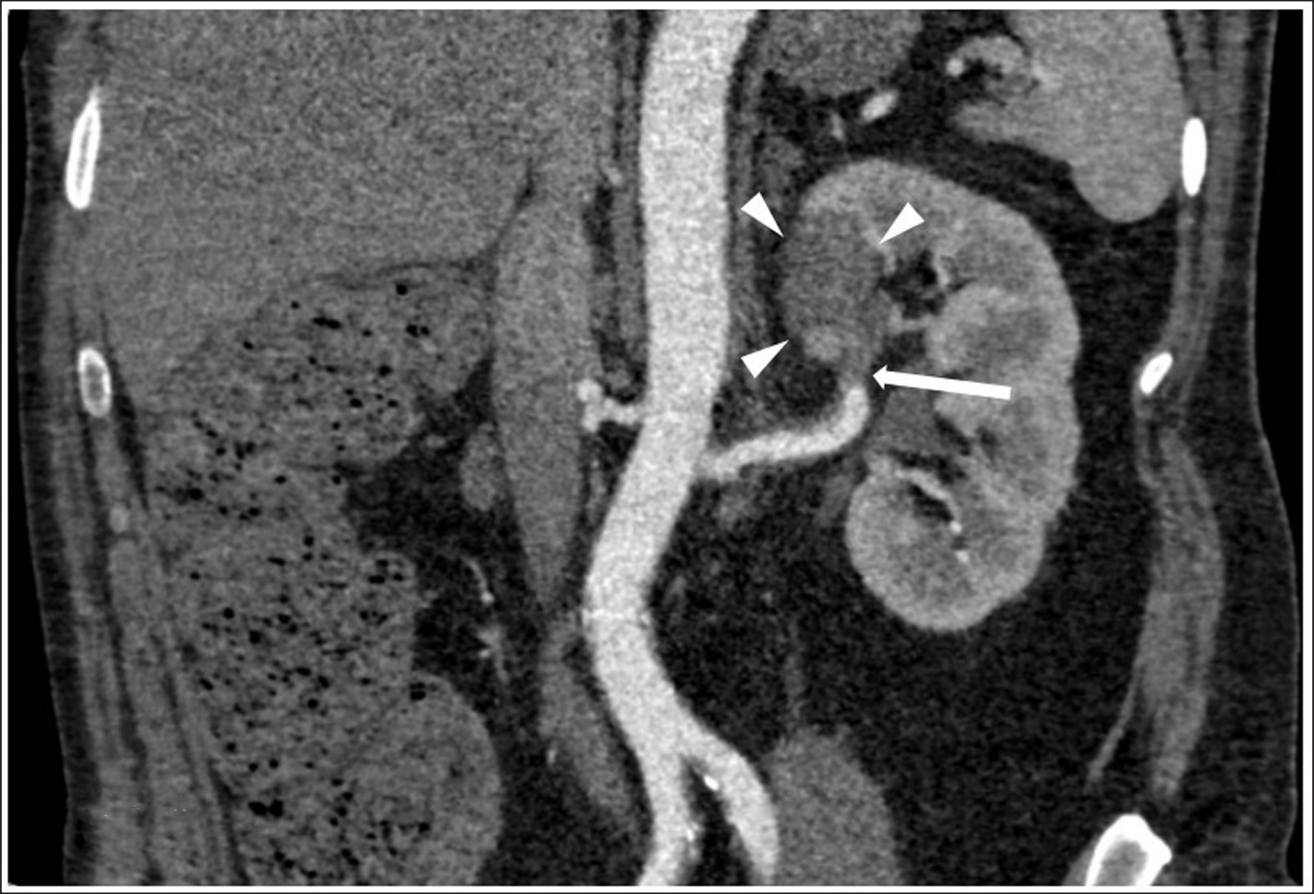


**Figure 2:**
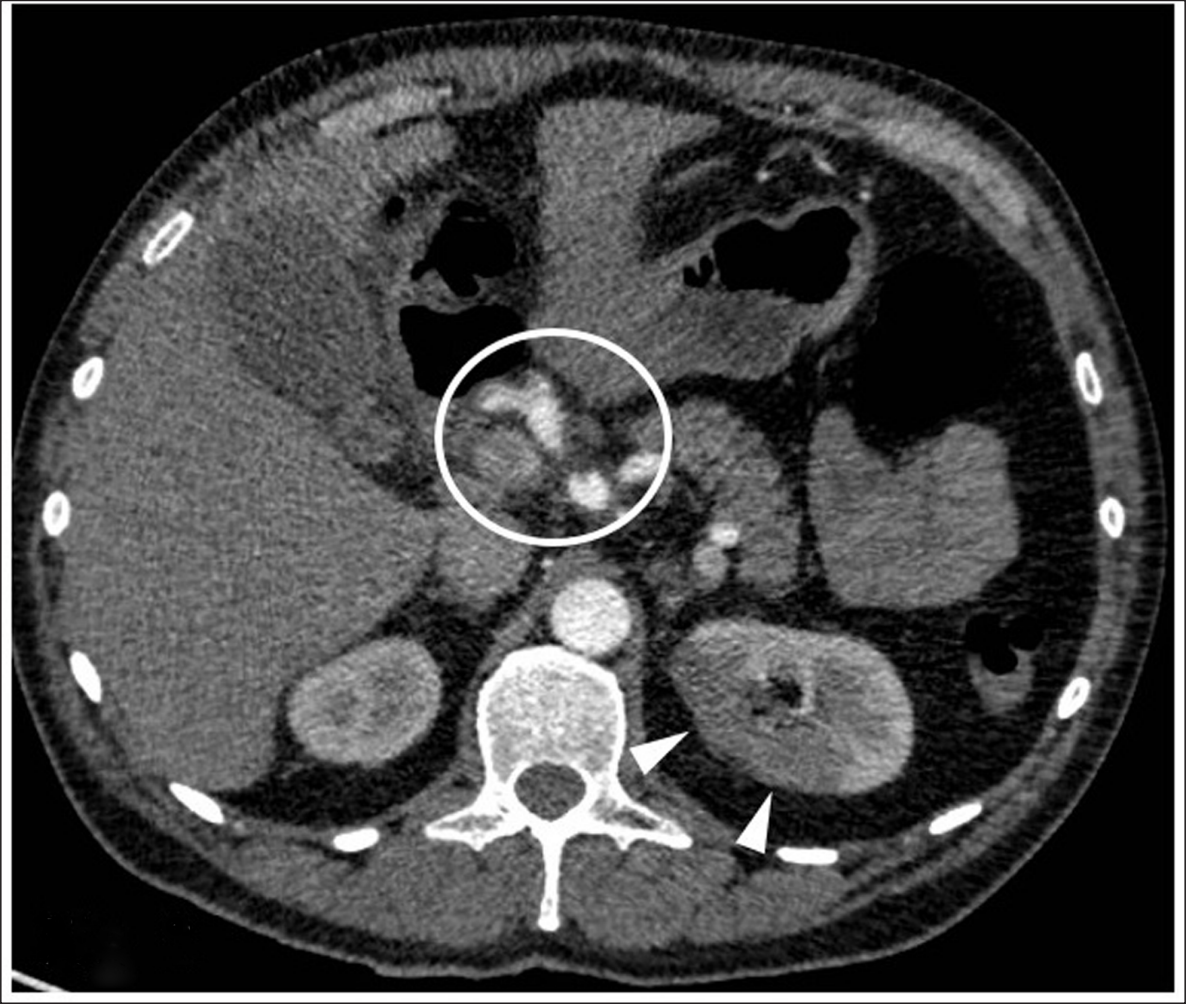


**Figure 3:**
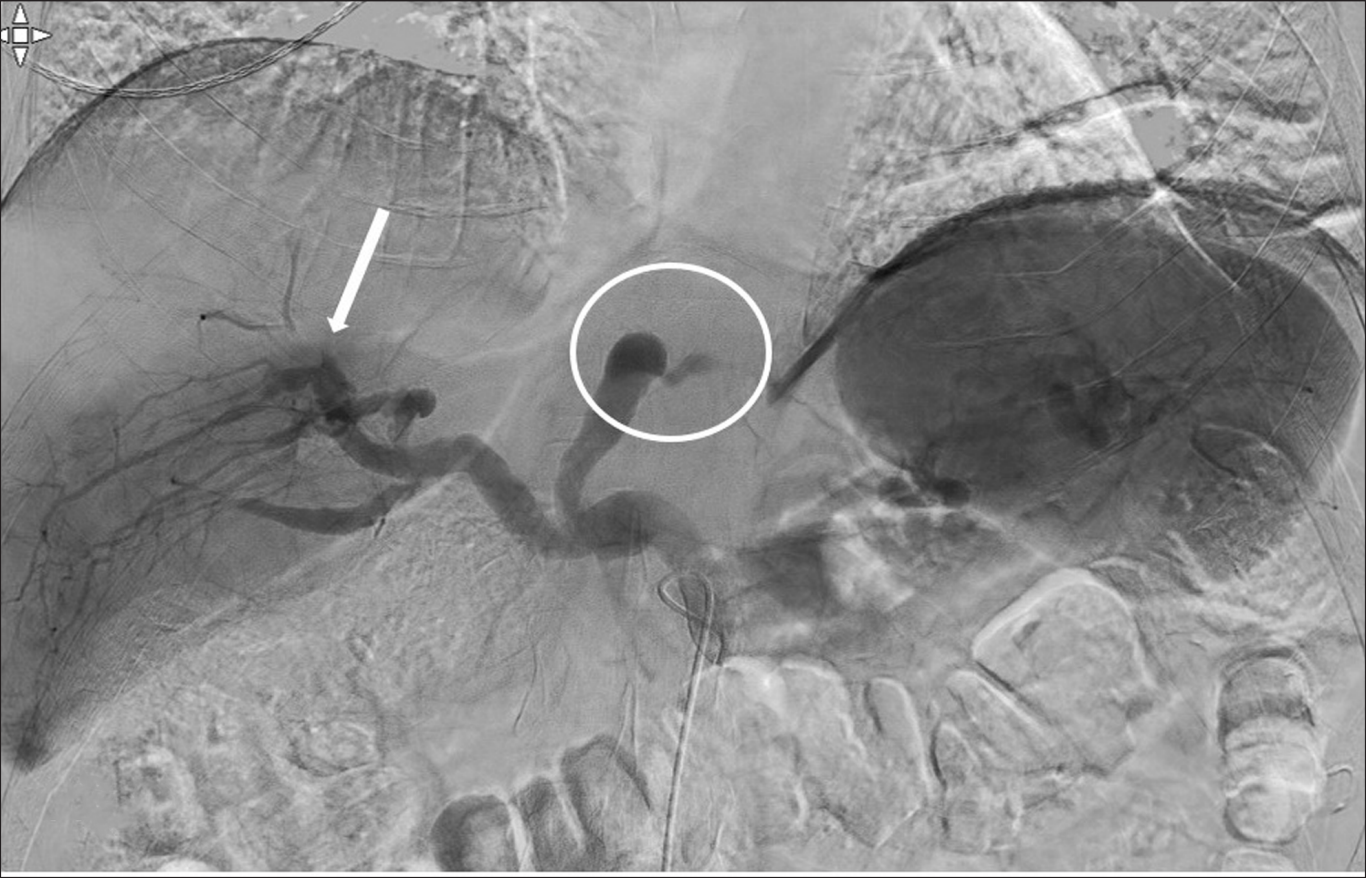


A 46-year-old male with unremarkable medical history presented with acute right flank pain. Contrast-enhanced abdominal computed tomography (CT) revealed a segmental right renal infarction (not shown). Three days later, similar complaints developed on the left side. CT angiography (***Figure 1*** curved planar reformat, ***Figure 2***, axial) showed a new segmental infarction in the left kidney upper pole (arrowheads) related to occlusion of the feeding segmental artery (arrow). In addition, a fusiform dilation and dissection of the coeliac trunk and common, right and left hepatic artery were found (circle). Digital subtraction angiography (DSA) six days after the initial presentation revealed occlusion of the left hepatic artery (***Figure 3***, circle) and cranial branches of the right hepatic artery (arrow) with absent arterial parenchymal opacification. DSA of the renal arteries ruled out (micro)aneurysm formation. In correlation with the clinical findings, the diagnosis of segmental arterial mediolysis (SAM) was made.

## Comment

SAM is a rare, non-inflammatory, non-atherosclerotic vasculopathy characterised by dissection and/or aneurysm formation in medium sized visceral arteries, associated with stenosis, occlusion, or rupture. Patients are typically male, aged between 40 and 60 years, presenting with acute abdominal pain due to organ ischemia or, less commonly, haemorrhage. SAM most commonly affects the renal arteries, superior mesenteric artery, coeliac trunk and hepatic artery. Iliac and splenic arteries are less commonly involved. In the majority of cases, two or more arteries are affected upon initial presentation [1].

The absence of contiguous aortic dissection, atherosclerosis, and features of hereditary connective tissue diseases (e.g. Marfan, Ehlers-Danlos) and fibromuscular dysplasia are important in the differential diagnosis. In the absence of dissection, differentiation with vasculitis is mainly based on laboratory findings. In this particular case, renal micro-aneurysms, which are associated with poly-arteritis nodosa, were ruled out with DSA.

Although disease stability or resolution is seen in most cases, follow-up imaging is recommended. Apart from clinical management of arterial dissection/occlusion with antiplatelets or anticoagulants, enlarging aneurysms should be endovascularly excluded.

## References

[1] Naidu SG, Menias CO, Oklu R, et al. Segmental arterial mediolysis: Abdominal imaging of and disease course in 111 patients. Am J Roentgenol. 2018; 210(4): 899 905. DOI: 10.2214/AJR.17.18309 29446669

